# The Journal in 1893

**Published:** 1894-01

**Authors:** 


### Editorial, Etc.

THE JOURNAL IN 1893.—One year ago we reviewed the Journal's record for 1892, and speaking for the year 1893 said under the head "Retrospect and Prospect": "For the coming year it is expected that additions will be made in the interest and value of the contents, and that the features will be so shaped as to be an aid to the dental practitioner. While during the year the JOURNAL has been made up partly of selections from other periodicals with only occasional original articles, and has been issued often three months behind date, yet it is gratifying to know that the contributions have been of general interest, and that the publishers have determined not to let the issue get behind again."

Respecting the fulfillment of those expectations, it is becoming in the first place to acknowledge the fidelity with which the publishers have met their promise to issue the JOURNAL during the current month, so that from January 1, 1893, to January 1, 1894, no issue has been behind its date. For this reason, among others the publishers should count with confidence upon the recognition of their efforts by the profession.

Some of the original articles in the JOURNAL in 1893 have been: "The World's Columbian Dental Congress;" "The First Pan-American Medical Congress;" "Uses of Electricity;" "Should Preachers Pay;" "Chapin A. Harris, M. D., D. D.S.;" "A Demonstration of Hypnotism;" "A New Lining for Vulcanite Plates;" "Diseased Breath;" "Progress of Therapeutics;" "The Progress of Surgery;" "Changes in the University of Maryland;" "Improvements in Dentistry;" "The Teeth as an Index of Civilization."

During 1893 we succeeded in reporting the doings, lectures and writings of leading dentists in this country and elsewhere, and in this way professional knowledge and experience have been made known to the readers of the JOURNAL.

No new features of special importance are in view for 1894.

